# The new evidence of China’s economic downturn: From structural bonus to structural imbalance

**DOI:** 10.1371/journal.pone.0257456

**Published:** 2021-09-23

**Authors:** Yimin Chen, Yulin Liu, Xin Fang

**Affiliations:** 1 School of Economics and Business Administration, Chongqing University, Chongqing, China; 2 Center for Public Economy & Public Policy Research, Chongqing University, Chongqing, China; 3 College of Business, Hawaii Pacific University, Honolulu, Hawaii, United States of America; School of Economics, Xiamen University, CHINA

## Abstract

The slowdown of China’s economic growth in the middle-income stage has caused widespread concerns. Based on the analysis of economic structures to explain the downward trend of economic growth, this study expanded the Solow-Swan model to investigate the structural imbalances and evaluated their impacts during the structural transformation in different stages and regions on the economic downturn. Considering the processes of production, distribution, and consumption, six structures were chosen for national and prefecture-levels in China from 1997 to 2017, including sectoral structure, population structure, investment and consumption structure, import-export structure, urban-rural income structure, and financial structure. The study found that China’s comprehensive economic structure was significantly different before and after the middle-income stage, and structural bonus tended to decline. Structural imbalance presented a U-shaped pattern of decreasing first and then increasing, and the impact on economic growth underwent stages of suppression-promotion-suppression. There was a significant difference in the imbalance of six sub-structures and their impacts; furthermore, in the four regions of east, center, west, and northeast the observations were very different. Taken together, the imbalance of economic structure and economic transformation coexisted, and the economic growth slowed down. Based on the experiences from China, this paper provided some evidence for promoting structural optimization and transformation.

## 1. Introduction

**Fig 1:**
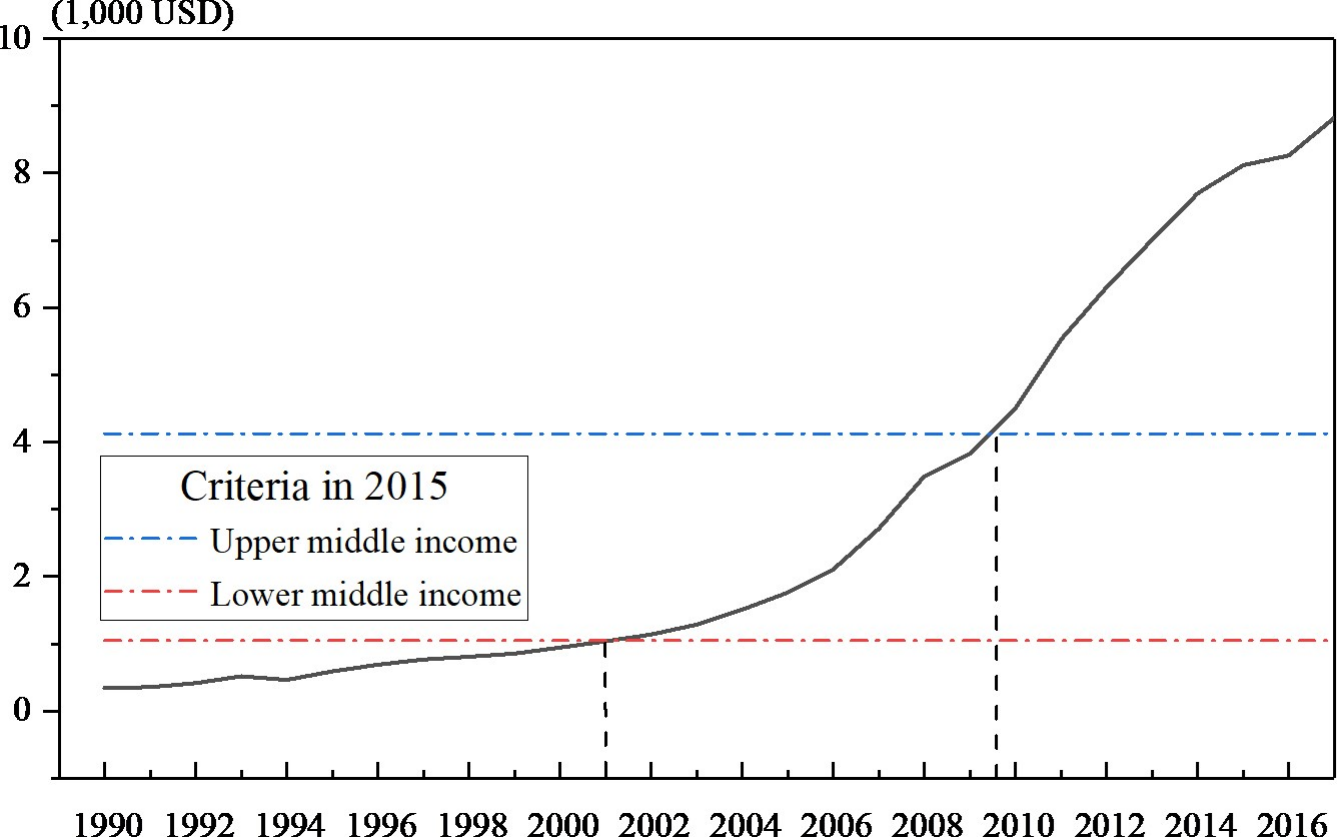
Per capita GNI of China (1990–2017).

**Fig 2:**
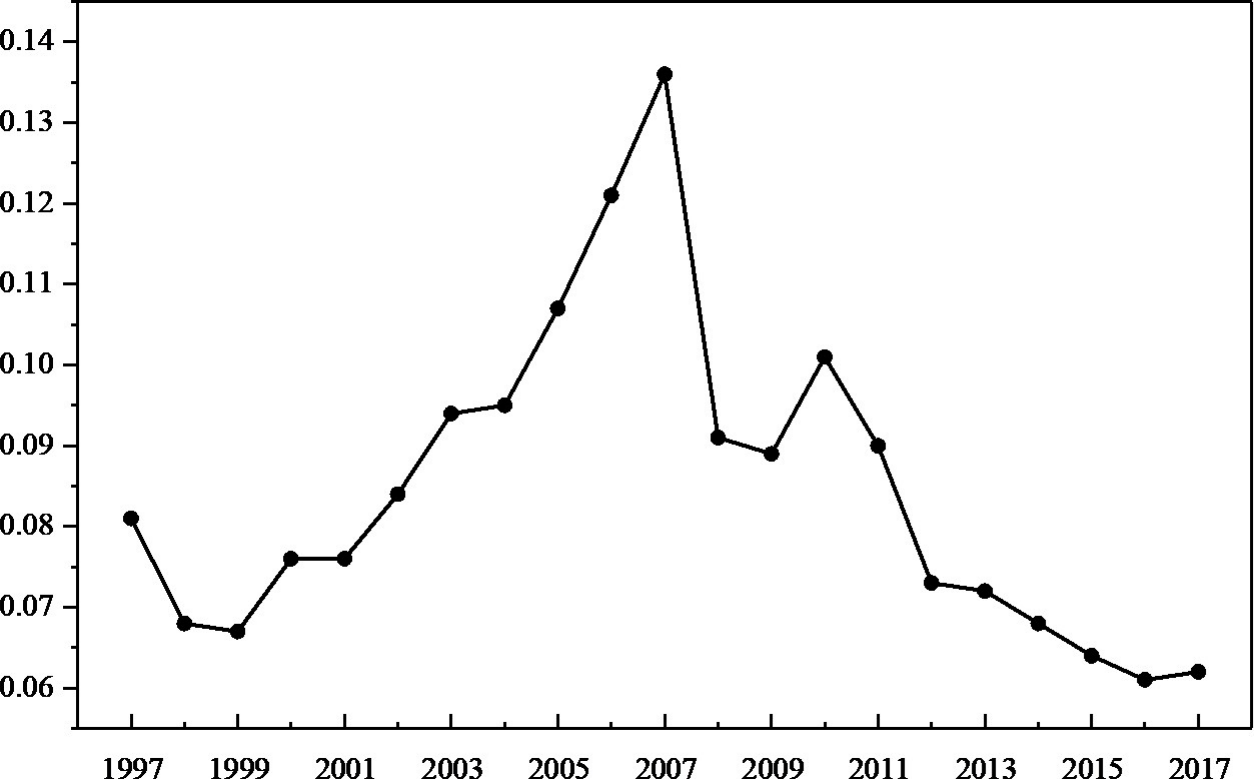
Real per capita GDP growth rate of China (1997–2017).

China has achieved rapid economic growth since the reform and opening up. According to the criteria of national income established by World Bank, China became a lower-middle-income country in 2001 and transformed to an upper-middle-income country in 2010 (Fig 1). However, China’s growth has been slowing down since 2010 (Fig 2). The academic debate about the “middle-income trap” has been very diverse. In the face of China’s economic downturn, the discussion on whether China falls into the “middle income trap” is even more prominent [1]. Whether or not a “trap” exists, cracking the mystery of the economic downturn black box has become an urgent research economic issue.

The economic practice of contemporary China shows that middle-income economies urgently need to explore a development momentum and development mechanism different from the low-income stage. Structural transformation has been argued to be an important aspect of economic development [2]. The upgrading and replacement will promote the rational coordination of economic structure to meet the needs of long-term development. Development issues at the middle-income stage can be attributed to the balance between economic structure and economic growth. After low-income economies enter the middle-income stage and transfer from a survival-oriented to a development-oriented society, the main conflicts of the economy and society at this time are transformed from the mismatch between aggregate demand and aggregate supply into a structural conflict.

Throughout the 70 years of economic development in China, the increase in total volume accompanied by structural imbalances is a typical feature. There is an inevitable transition from the structural acceleration in the industrialization phase to the structural deceleration in the urbanization phase [3]. After entering the middle-income stage, economic growth is affected by slower changes in economic development patterns and constrained by the relatively lagged economic reforms. The global economic crisis that broke out in 2008 led to a slowdown in economic growth and made the structural imbalances of China’s economy more complicated. After entering the New Era, with a new historic juncture in China’s development proposed by Xi Jinping, the main conflict in Chinese society has been transformed. Moreover, structural imbalances threaten China’s economic growth potential and affect China’s economic development quality.

The economic structure is always the focus of academic communities, and the Chinese government pays increasing attention to its importance. The report of the 17th National Congress of the Communist Party of China (http://www.china.com.cn/policy/txt/2007-10/24/content_9435992_5.htm) pointed out that China should vigorously push forward strategic economic restructuring, accelerate the transformation of the mode of economic development and promote the upgrade of sectoral structure. The report of the 18th National Congress (http://www.xinhuanet.com//18cpcnc/2012-11/17/c_113711665.htm) pointed out that carrying out strategic adjustment of the economic structure was the major goal of accelerating the change of the growth model. China should strive to remove major structural barriers to achieving sustainable and sound economic development, with a focus on improving demand structure and sectoral structure, promoting co-development across regions, and advancing urbanization. The understanding of economic structure has changed from focusing on sectoral structure only to much broader aspects. The report of the 19th National Congress (http://www.china.com.cn/cppcc/2017-10/18/content_41752399.htm) stressed that it was a pivotal stage for transforming China’s growth model, improving economic structure, and fostering new drivers of growth. China should pursue supply-side structural reform as the main task, with an emphasis on better quality, higher efficiency, and more robust drivers of economic growth through reforms. Therefore, study of the relationship between economic structure and economic growth might provide a more comprehensive understanding of China’s economic development.

For a long time, sectoral structure has been examined comprehensively, but as a narrowly defined economic structure [4]. The economic structure is defined as sectoral structure that focuses on economic scale and productivity development [5]. In the early stage of economic evolution, the production scale and sectoral structure play the major role [6]. Sectoral structure is measured by the output share and labor share of each sector [7], the specialization, diversity and competition of each sector [8, 9]. With further investigations, some scholars pointed out the insufficiency of traditional sectoral structure research and conducted more general studies on the economic structure. The new definition includes labor structure, international trade structure, and financial structure, etc. The research has been extended to resource allocation, income distribution, investment, urbanization, capital, foreign exchange, and stocks [2, 10–12]. To better measure the economic structure, economic complexity was suggested which uses lots of sub-structure factors [6, 13–14].

There exists a large literature in the field of development economics discussing changes in the economic structure [15, 16]. However, most of them focus on the interpretation of, phenomena and the analysis of results. Measures of structural development have not received much attention. For the relationship between economic structure and economic growth, the “structural bonus hypothesis” becomes the focus of research [17]. The follow-up studies categorize the contribution of sectoral structural change to the productivity growth due to the movements of factors of production as “structural bonus hypothesis” [18]. Some studies qualitatively prove the mechanism of economic (sectoral) structure affecting economic growth of various sectors by constructing theoretical research [7, 9]. Other studies quantitatively evaluate the contribution of economic (sectoral) structure to economic growth through empirical tests [8, 11, 19].

Up to now, there is no unified standard for the connotation of economic structure or the development, evolution, and imbalance of economic structure. The studies of the relationship between economic structure and economic growth do not come into a systematic theory. To address this issue, this paper took national level and 286 prefecture-levels data from 1997 to 2017 as empirical objects and examined the structural imbalances and their impacts on different stages and regions. In particular, the following questions were evaluated: First, was there an imbalance in China’s economic structure? How did the economic structure affect economic growth? Were there differences across regions and stages? Second, was China facing the risk of economic stagnation caused by imbalanced economic structure? Third, what kind of policy support is needed for the transformation of the economic structure and the transition to break through the middle-income stage? This paper explained the slowdown of China’s economic growth based on the structural perspective; expanded the generalized economic structure and measured structural imbalances; and gave a systematical framework to research economic structure and economic growth.

The rest of this paper was organized as follows. The second part provided the internal logic of economic structure and economic growth; the third part described the variables and explained data; the fourth part presented the identification and test of economic structure and economic growth; the fifth part was the robustness test; the sixth part drew the conclusion.

## 2. The internal logic of economic structure and economic growth

This section followed the general laws of economic phenomena, set hypothetical conditions, constructed ideal models of economic structure, economic growth, and its impact mechanisms, deconstructed the black box of economic growth, explained objective economic phenomena under realistic conditions, and recommended a path consistent with Chinese reality.

### 2.1 The path of economic growth in different stages under ideal conditions

The theoretical model mainly emphasized the following two aspects: to describe how changes of economic structure affect economic growth; to reflect how different income stages affect economic growth. Therefore, we considered a production function model that reflected the changes of economic structure and its production factors. Inspired by the ideas of Kongsamut et al. [7], this section extended the Solow-Swan Model.

The model uses the rate of technological progress to indicate the level of contribution of technological factors to economic growth [20–22]. Increasing the rate of technological progress can greatly improve production efficiency and output levels, which is an important way to achieve leapfrog development. Driven by technological progress, resources and factors are shifting from low-productivity sectors to high-productivity sectors, so that high-productivity sectors are given priority in their development, low-productivity sectors are constantly being eliminated and phasing out, and finally the economic structure is optimized and upgraded. Bulman et al. found that the increase in technological progress is the source of economic growth [1]. Due to physical capital accumulation restrictions and diminishing marginal returns to capital, the leapfrog development from low-income to middle-income to high-income stages can only be achieved through education, research and development, reform, and innovations.

Based on the Solow-Swan model, the basic hypothesis of the theoretical model is put forward: the economy uses two production factors, labor and capital, for production. In the low-income stage, production sectors include traditional agriculture, manufacturing, and services. The traditional agriculture does not employ capital and it only requires basic labor skills to produce essential necessities. The development of manufacturing industries promotes the transition from the low-income stage to the middle-income stage. Agricultural technologies start to develop and capital enters agriculture. The production sectors transform into traditional agriculture, modern agriculture, manufacturing, and services after entering the middle-income stage. Ultimately, the traditional agriculture is replaced completely, and the production sectors transform into modern agriculture, manufacturing, and services. Therefore, the theoretical model consists of four sectors: traditional agriculture (0), modern agriculture (1), manufacturing (2), and services (3); three products: agricultural products (1), manufactured products (2), and services (3) (including financial services and business services [23]); two factors of production: labor (*L*) and capital (*K*).

#### Production

Suppose that an economic agent (regardless of gender) is born at time *t*, lives through the period [*t*,*t*+1), and dies after giving birth to a descendant at time *t*+1. Then the population size remains unchanged. Suppose that labor supply is perfectly inelastic, so we normalize the total amount of labor available in the economy to 1 at every point in time. Suppose that the production function takes Cobb-Douglas form, then the production functions of the four sectors are as follows.


$$Y_{0,t}=B_{0}L_{0,t}$$ (1)

$$Y_{1,t}=B_{1}{\left(\phi_{1,t}K_{t}\right)}^{\alpha }{\left(A_{t}L_{1,t}\right)}^{1-\alpha }$$ (2)

$$Y_{2,t}=B_{2}{\left(\phi_{2,t}K_{t}\right)}^{\alpha }{\left(A_{t}L_{2,t}\right)}^{1-\alpha }$$ (3)

$$Y_{3,t}=B_{3}{\left(\phi_{3,t}K_{t}\right)}^{\alpha }{\left(A_{t}L_{3,t}\right)}^{1-\alpha }$$ (4)

Where the variables *B*_*i*_, *ϕ*_*i*,*t*_, and *A*_*t*_ denote, respectively, relative productivity proportionality, the fraction of capital, and the level of exogenous technological progress; and $\frac{A_{t+1}}{A_{t}}=1+g(A_{t}>0,g>0)$.

#### Consumption

Suppose that the economic agent maximizes life-time total utility and the preferences are time-separable. The momentary utility function is as follow.


$$U=\int_{0}^{\infty }e^{-\rho t}\frac{[{\left(C_{1,t}-{\bar{C}}_{1}\right)}^{\gamma_{1}}C_{2,t}^{\gamma_{2}}{\left(C_{3,t}+{\bar{C}}_{3}\right)}^{\gamma_{3}}]-1}{1-\sigma }dt$$ (5)

Where the variables ${\bar{C}}_{1}$, ${\bar{C}}_{3}$, *ρ*, *σ* and *γ*_*i*_ denote, respectively, the level of subsistence consumption for agricultural products, the household (non-market) production of services, discount rate, relative risk aversion factor, and income elasticity of product demand ($\sum_{i=1}^{3}\gamma_{i}=1$).

#### Market equilibrium

When the competitive market reaches equilibrium, the conditions for market clearing are as follows.


$$Y_{0,t}+Y_{1,t}=C_{1,t}$$ (6)

$$Y_{2,t}=C_{2,t}+{\dot{K}}_{t}+\delta K_{t}$$ (7)

$$Y_{3,t}=C_{3,t}$$ (8)

$$\sum_{i=0}^{3}L_{i,t}=1$$ (9)

$$\sum_{i=1}^{3}\phi_{i,t}=1$$ (10)

Where the variable *δ* denotes capital depreciation rate. $\left({\dot{K}}_{t}+\delta K_{t}\right)$ denotes investment.

Since capital and labor are mobile freely across sectors 1, 2, and 3. Therefore, the marginal rate of transformation across the three sectors is equal. The following equation can be derived.


$$\frac{\phi_{1,t}}{L_{1,t}}=\frac{\phi_{2,t}}{L_{2,t}}=\frac{\phi_{3,t}}{L_{3,t}}=\frac{1}{1-L_{0,t}}$$ (11)

And the per capita capital is the same.


$$k_{i,t}=\frac{\phi_{i,t}K_{t}}{A_{t}L_{i,t}}=\frac{K_{t}}{A_{t}(1-L_{0,t})}\equiv k_{t}(i=1,2,3)$$ (12)

Marginal outputs of labor (which are in inverse proportion to the product prices), i.e., wage rates are as follows.


$$w_{0}=\frac{\partial Y_{0,t}}{\partial L_{0,t}}=B_{0}$$ (13)

$$w_{i}=\frac{\partial Y_{i,t}}{\partial L_{i,t}}=B_{i}(1-\alpha )k_{t}^{\alpha }A_{t}(i=1,2,3)$$ (14)

Marginal output of capital, i.e., interest rate is as follow.


$$r_{t}=\frac{\partial Y_{2,t}}{\partial \phi_{2,t}K_{t}}=B_{2}\alpha k_{t}^{\alpha -1}$$ (15)

The following equation can be derived from (15).


$$k_{t}={\left(\frac{r}{B_{2}\alpha }\right)}^{\frac{1}{\alpha -1}}$$ (16)

The relative price of various products for manufactured products are as follows.


$$p_{0,t}=\frac{P_{0,t}}{P_{2,t}}=\frac{B_{2}}{B_{0}}(1-\alpha )k_{t}^{\alpha }A_{t}$$ (17)

$$p_{1,t}=\frac{P_{1,t}}{P_{2,t}}=\frac{B_{2}}{B_{1}}\equiv p_{1}$$ (18)

$$p_{3,t}=\frac{P_{3,t}}{P_{2,t}}=\frac{B_{2}}{B_{3}}\equiv p_{3}$$ (19)

Using the relative price, the production constraint expressed in manufacturing can be written as

$$p_{1}C_{1,t}+C_{2,t}+{\dot{K}}_{t}+\delta K_{t}+p_{3}C_{3,t}=B_{2}K_{t}^{\alpha }{\left[A_{t}(1-L_{0,t})\right]}^{1-\alpha }=B_{2}k_{t}^{\alpha }A_{t}(1-L_{0,t})$$ (20)

The consumption distribution can be written as

$$\frac{p_{1}(C_{1,t}-{\bar{C}}_{1})}{\gamma_{1}}=\frac{C_{2,t}}{\gamma_{2}}=\frac{p_{3}(C_{3,t}+{\bar{C}}_{3})}{\gamma_{3}}$$ (21)

According to the relevant conditions of market clearing above, the balanced evolution process of different income stages can be revealed:

#### Low-income stage: (0+2+3) mode

Low-income economies are subsistence societies. The production sector includes traditional agriculture, manufacturing and services. Traditional agriculture does not include capital. It uses only basic skills to produce subsistence agricultural products and it is a closed economy without trade.

The agricultural labor at this time is

$$L_{0,t}=\frac{{\bar{C}}_{1}}{B_{0}}\equiv {\bar{L}}_{0}$$ (22)

Per capita output of agriculture is

$$\frac{p_{0,t}{\bar{C}}_{1}}{{\bar{L}}_{0}}=B_{2}(1-\alpha )k_{t}^{\alpha }A_{t}$$ (23)

Per capita output of modern sector is

$$\frac{B_{2}k_{t}^{\alpha }A_{t}(1-{\bar{L}}_{0})}{1-{\bar{L}}_{0}}=B_{2}k_{t}^{\alpha }A_{t}$$ (24)

Comparing (23) and (24) we can draw the Conclusion I: The per capita output of agricultural sector in low-income economies is lower than the per-capita output of the non-agricultural sector. The development of the non-agricultural sector is the main driving force for the economy to move out of the low-income stage.

#### Middle-income stage: (0+1+2+3) mode

The development of the non-agricultural sector promoted the transition from the low-income stage to the middle-income stage, and the economy shifted to a developing society. Agricultural technology began to transform, capital entered agriculture, and the production sector was transformed into traditional agriculture, modern agriculture, manufacturing, and services, and gradually became an open economy.

As agricultural technology transforms, capital enters agriculture and *k*_*t*_ begins to decrease. Suppose that at the time *q*, traditional agriculture and modern agriculture have the same price and reach equilibrium.


$$\frac{P_{0,t}}{P_{1,t}}=\frac{P_{0,t}}{P_{2,t}}\frac{P_{2,t}}{P_{1,t}}=\frac{B_{1}}{B_{0}}(1-\alpha )k_{t}^{\alpha }A_{t}=1$$ (25)

The following equation can be derived from (25)

$$k_{q}={\left[\frac{B_{0}}{B_{1}(1-\alpha )A_{t}}\right]}^{\frac{1}{\alpha }}$$ (26)

The total output in the low-income stage is given by (20): $B_{2}k_{t}^{\alpha }A_{t}(1-L_{0,t})$.

The total output at time *q* and beyond is

$$B_{2}k_{q+}^{\alpha }A_{t}(1-L_{0,q+})$$ (27)

Comparing (20) and (27) we can see that in order to ensure the growth of total output, *L*_*0*,*q+*_ has to decline. Therefore, we get the Conclusion II: after entering the middle-income stage, economic growth will inevitably be accompanied by a decrease in traditional agricultural employment, and technological progress will become the driving force for sustained growth.

#### Generalized Balanced Growth Path: (1+2+3) mode

Traditional agricultural employment continued to decline. Eventually, traditional agriculture was replaced by modern agriculture, and the production sector was transformed into modern agriculture, manufacturing, and services. At the same time, as the degree of openness increased, the trade structure became more closely linked to its economy [24].

In an open economy, products can be traded internationally, and capital and labor can move freely between economies. Therefore, the world’s relative prices, *p*_i_*, world interest rates, r*, and world wage rates, *w*_i_*, are exogenously determined [25]. Following the constraints of Kongsamut et al. [7], we assume 1. $\frac{{\bar{C}}_{1}}{{\bar{C}}_{3}}=\frac{B_{1}}{B_{3}}$; 2. r* = *gσ*+*ρ*, and derive a Generalized Balanced Growth Path for the middle-income stage.

#### Domestic market

(20) can be rewritten as

$$p_{1}(C_{1,t}-{\bar{C}}_{1})+C_{2,t}+{\dot{K}}_{t}+\delta K_{t}+p_{3}(C_{3,t}-{\bar{C}}_{3})=B_{2}k_{t}^{\alpha }A_{t}$$ (28)

The growth rates of labor in three sectors are as follows.


$$\frac{{\dot{L}}_{1,t}}{L_{1,t}}=-g\frac{{\bar{C}}_{1}}{B_{1}\phi_{1,t}K_{t}}$$ (29)

$$\frac{{\dot{L}}_{2,t}}{L_{2,t}}=0$$ (30)

$$\frac{{\dot{L}}_{3,t}}{L_{3,t}}=g\frac{{\bar{C}}_{3}}{B_{3}\phi_{3,t}K_{t}}$$ (31)

The growth rates of output (given by consumption in market equilibrium) in three sectors are as follows.


$$\frac{{\dot{C}}_{1,t}}{C_{1,t}}=g\frac{C_{1,t}-{\bar{C}}_{1}}{C_{1,t}}$$ (32)

$$\frac{{\dot{C}}_{2,t}}{C_{2,t}}=g$$ (33)

$$\frac{{\dot{C}}_{3,t}}{C_{3,t}}=g\frac{C_{3,t}+{\bar{C}}_{3}}{C_{3,t}}$$ (34)

#### Imports and exports

We modified (28) as production constraints are determined by world prices:

$$p_{1}^{*}(C_{1,t}^{*}-{\bar{C}}_{1})+p_{2}^{*}C_{2,t}^{*}+p_{3}^{*}(C_{3,t}-{\bar{C}}_{3})=B_{2}k_{t}^{\alpha }A_{t}-({\dot{K}}_{t}+\delta K_{t})=C_{D,t}+X_{t}$$ (35)

Where, *C*_*D*,*t*_ denotes domestic aggregate demand, *X*_*t*_>0 denotes exports, *X*_*t*_<0 denotes imports.

The imports/exports volume can be derived:

$$X_{t}=\sum_{i=1,2,3}\frac{p_{i}^{*}-p_{i}}{p_{i}^{*}p_{i}}B_{i}[B_{2}k_{t}^{\alpha }A_{t}-({\dot{K}}_{t}+\delta K_{t})]$$ (36)

Comparing (29)–(34) we can see that $-g\frac{{\bar{C}}_{1}}{B_{1}\phi_{1,t}K_{t}}<0<g\frac{{\bar{C}}_{3}}{B_{3}\phi_{3,t}K_{t}}$; $g\frac{C_{1,t}-{\bar{C}}_{1}}{C_{1,t}}<g<g\frac{C_{3,t}+{\bar{C}}_{3}}{C_{3,t}}$. Therefore, we reach the Conclusion III: continuing to reduce agricultural employment, stabilize industrial employment, and increase employment in the service industry would be the inevitable path for the economy to reach a Generalized Balanced Growth Path at the middle-income stage. At this stage, industrial output growth is higher than agricultural output growth but lower than service output growth. The Conclusion IV can be derived from (36): in an open economy, economies face import and export choices affecting by world product prices and they can adjust the production of various domestic sectors to adapt to trade impacts.

Low-income stage, Middle-income stage and Generalized Balanced Growth Path are link to each other endogenously, and they are different phases of economic development. The derived conclusions of the above three models are consistent with the “structural bonus hypothesis” proposed by Timmer and Szirmai [18]. Therefore, the following theoretical proposition is obtained:

The balanced evolution of the economic structure has reconfigured the structural elements, stimulated the structural bonus, promoted the realization of the Generalized Balanced Growth Path, and had a positive impact on the economy.

### 2.2 Theoretical mechanism analysis of China’s economic structure and economic growth

The theoretical model takes the narrow economic structure (sectoral structure) as the entry point to describe the evolution of economic structure from the low-income stage to the middle-income stage and finally to reach the Generalized Balanced Growth Path. Although the general equilibrium path can reveal the evolution law of economic growth at different development stages, the economic growth of each country has its unique evolution path.

**Fig 3:**
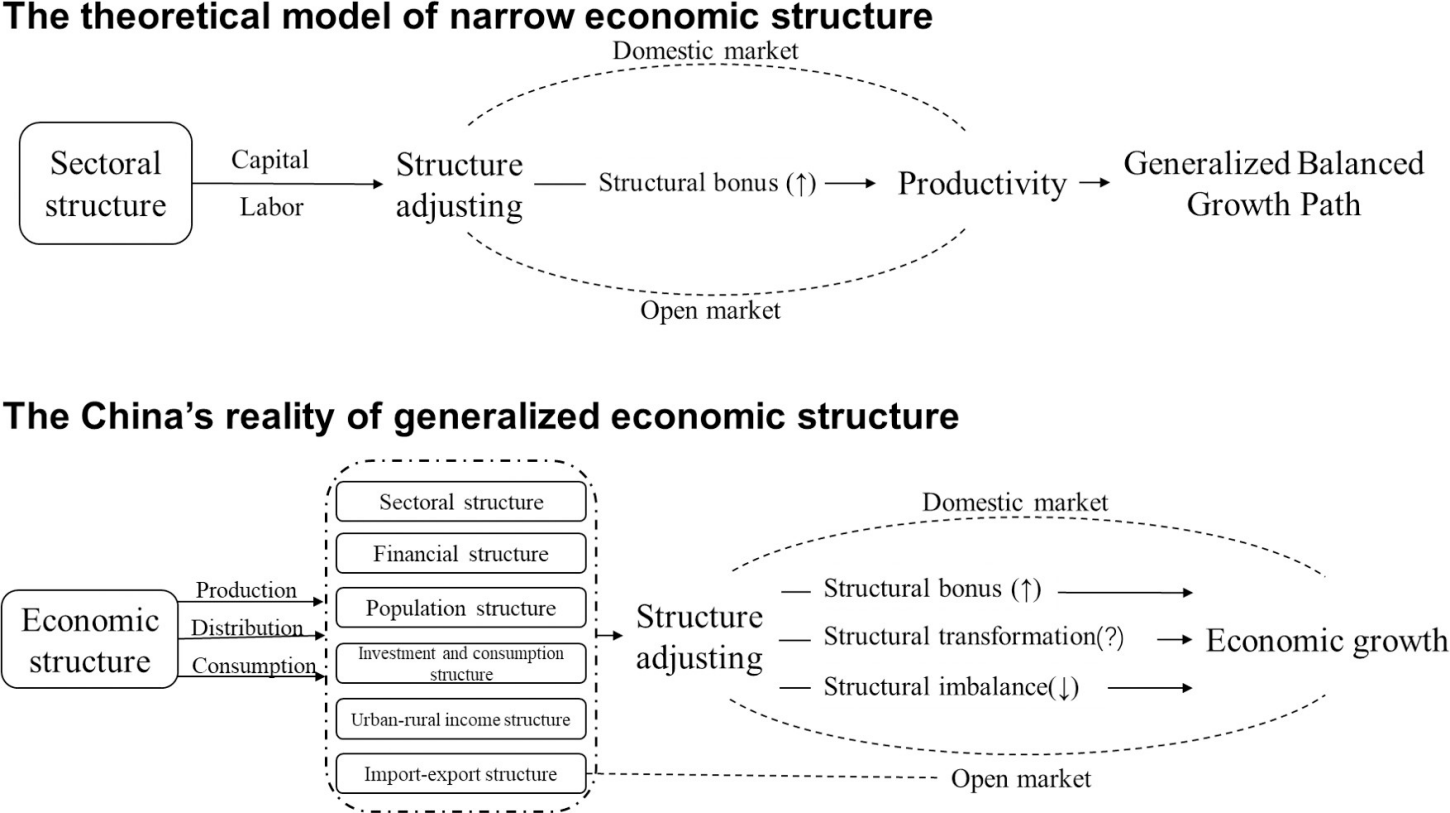
The framework of structural relationship path.

Based on the theoretical model and existing literature, this paper expanded the generalized economic structure into six aspects including sectoral structure, population structure, investment and consumption structure, import-export structure, urban-rural income structure, and financial structure. We built a framework of structural relationship path (Fig 3) to clarify the inherent logic of economic structure and economic growth.

The theoretical model depicts economic growth from the perspective of production, distribution, and consumption. From the perspective of production, sectoral structure is used as the starting point, focusing on the allocation of production factors. Among them, labor relates to population structure, and capital relates to investment and consumption structure as well as financial structure. The distribution perspective involves the income structure of urban and rural areas; the perspective of consumption involves the investment and consumption structure; and also considers structure of imports and exports in an open economy. The following will explain the impact mechanism of the structures on China’s economy.

For developing countries, the migration of labor as the main factor of production from the agricultural sector to the industrial sector increases productivity. Furthermore, industrialization promotes urbanization, and labor is transferred from the industrial sector to the service sector [2, 26]. During the industrialization period, sectoral structure of China’s sustained and accelerated economic growth is due to the higher industrial growth rate and the improvement of industry-led efficiency [3]. The industrialization expansion and corresponding efficiency improvements originated from “Learning by Doing” effect [27]. However, with the end of the large-scale industrialization phase, nevertheless, the acquisition of technology gradually gets harder. With the rapid progress of urbanization, the tertiary industry plays an increasingly important role in sectoral structure, and “servicizing” becomes a typical fact of economic development [23]. A favorable population structure can lead to a higher labor growth rate and an effective re-allocation in the industries, forming the “demographic dividend” [3]. As the stage of population development changes, the demographic dividend appears to decline or even be depleted. After entering the middle-income stage, China is facing an increasingly serious problem of aging population and labor. However, per capita income is still low. The demographic dividend is on the verge of disappearing and population structure will continue to age on average in the coming decades. China now finds itself in a challenging position of getting old before getting rich [28, 29]. The coexistence of high investment rate and high saving rate is a reality in China. A selective high investment leads to excessive growth in some industries, creating unproductive investments. The excess supply of labor makes it necessary for employees who enter the emerging sector to adapt to the new competition and become net savers with a low level of current consumption [30]. With the changes of sectoral structure and population structure, economy servicizing becomes prominent and demographic dividend exhausts gradually, and the imbalance of investment and consumption structure in economy begins to intensify.

Overcapacity and domestic under-consumption make excess supply indigestible, and the economy seeks external demand, which is reflected in the increase of the trade surplus. The development of international trade promotes the accumulation of human capital in the process of labor mobility and improves the efficiency of specialized production [31]. However, as the trade surplus increases significantly, large-scale foreign exchange reserves accumulate, and the dependence on the international market becomes increasingly serious. At the same time, large trade surpluses also lead to increased international trade frictions, increased pressure for currency appreciation, and excess domestic money supply. Impacted by the global economic crisis and the trade disputes between China and the United States, the Chinese economy is facing great challenges. The income gap affects potential output by affecting factors of production, which in turn affects economic growth rates and levels of economic growth [32]. The accumulation of physical capital is the main source of economic growth when the economy is small. The expansion of the income gap is conducive to the accumulation of physical capital, and an appropriate income gap is conducive to economic growth. Human capital plays a leading role in promoting economic growth when the size of economy grows large. The expansion of the income gap restricts the increase of human capital investment by low-income class [33]. In the early years of China’s reform and opening-up, an appropriate urban-rural income gap contributes to the accumulation and investment of physical capital, and weakens the constraints of supply. However, with the rapid economic and the relatively abundant accumulation of material capital, economic growth depends more on technological progress and the increase of human capital. The urban-rural income gap constrains the advancement of agricultural technology and the improvement of rural human capital. On the other hand, the urban-rural income gap cannot drive up the demand of rural residents, thus increasing the deficiency of residential demand.

Regarding the impact of financial structure on economic growth, the main focus of debate in the academic circle is the choice between the bank-oriented financial system and the market-oriented financial system. Some studies emphasize the advantages of banks in terms of savings mobilization, project selection, corporate supervision, and risk management [34–36]. Other studies support the positive role of markets in terms of information disclosure, technological advancement, management improvement, and risk dispersion [37–39]. Liu and Zhang report that there exists an optimal financial structure evolving to meet the different demands of the real economy in the process of economic development [40]. These studies solved the so-called “paradox” and focus on the practical effect of financial function on economic growth. Financial structure affects asset prices, thereby affecting sectoral structure and the investment and consumption structure, and exacerbates the imbalance of the real economic structure. At the same time, the unbalanced development of international trade also puts financial structure at risk of imbalance.

## 3. Data

China joined the group of lower-middle-income and upper-income countries in 2001 and 2010, so the period from 1997 to 2017 was selected as the research interval to cover 2001 and 2010.

There are several databases used in this paper. CEIC China Premium Database covers gross domestic product, population, Num. of employee, total & average wage, disposable income per capita, consumption expenditure per capita, export, import, loan, saving deposit, government revenue, government expenditure, fixed asset investment, foreign direct investment, the land area of administrative zone, and other economic variables (https://www.ceicdata.com). We retrieved missing values in some years from the National Bureau of Statistics Database of China covers national (http://www.stats.gov.cn/), China Economic and Social Big Data Research Platform of CNKI (http://www.stats.gov.cn/), and China City Statistical Yearbook-2017. The Global Financial Development Database 2017 of Credit & Financial Development Division (CFDD) is a global database, which covers stock market capitalization to GDP, Stock market total value traded to GDP, outstanding domestic private debt securities to GDP, Bank deposits to GDP, loans from nonresident banks to GDP, and other financial variables of 214 countries or regions (http://nacm.org/welcome-to-cfdd.html).

The data was used to construct the “aggregate China indicators”. Based on the above indicators and simplification upon the availability of data, we used the prefecture-level panel data to build “prefecture-levels indicators”, as shown in Table 1. Note that all variables above were standardized. See S1 File for a detailed explanation of data processing.

**Table 1:** Data list.

Indicators	Aggregate China	Prefecture-levels
Sectoral structure (*ss*)	Industry comparison index	Economy servicizing index
	Economy servicizing index	
	Unit GDP energy consumption	
Population structure (*ps*)	Children’s dependency ratio	Total population participation rate
	Elderly dependency ratio	
	Gini coefficient of education	
	Total population participation rate	
Investment and consumption structure (*ic*)	Investment rate	Ratio of investment to consumption
	Consumption rate	
	Ratio of investment to consumption	
Import-export structure (*ie*)	Trade surplus ratio	Ratio of export to import
	Total trade ratio	
	Foreign exchange reserve ratio	
Urban-rural income structure (*ur*)	Theil index of urban-rural income gap	Ratio of urban income to rural
	Ratio of urban Engel index to rural	
Financial structure (*fs*)	Ratio of direct financing to indirect	Loan ratio
	M2 ratio	
Comprehensive structure (*cos*)	Synthesized by principal component analysis method	Synthesized by weighted average method
Economic growth (*lnrpgdp*)	Napierian logarithm of real per capita GDP	

## 4. Identification and test of economic structure and economic growth

### 4.1 Dynamic identification of economic structure in different stages

Factor scores for the aggregate China’s economic structure development from 1997 to 2017 were obtained using Principal Component Analysis. We used this indicator and the Napierian logarithm of real per capita GDP, adopting the idea of Regression Discontinuity Design to analyze China’s economic structure development and economic growth after entering the middle-income stage.

**Fig 4:**
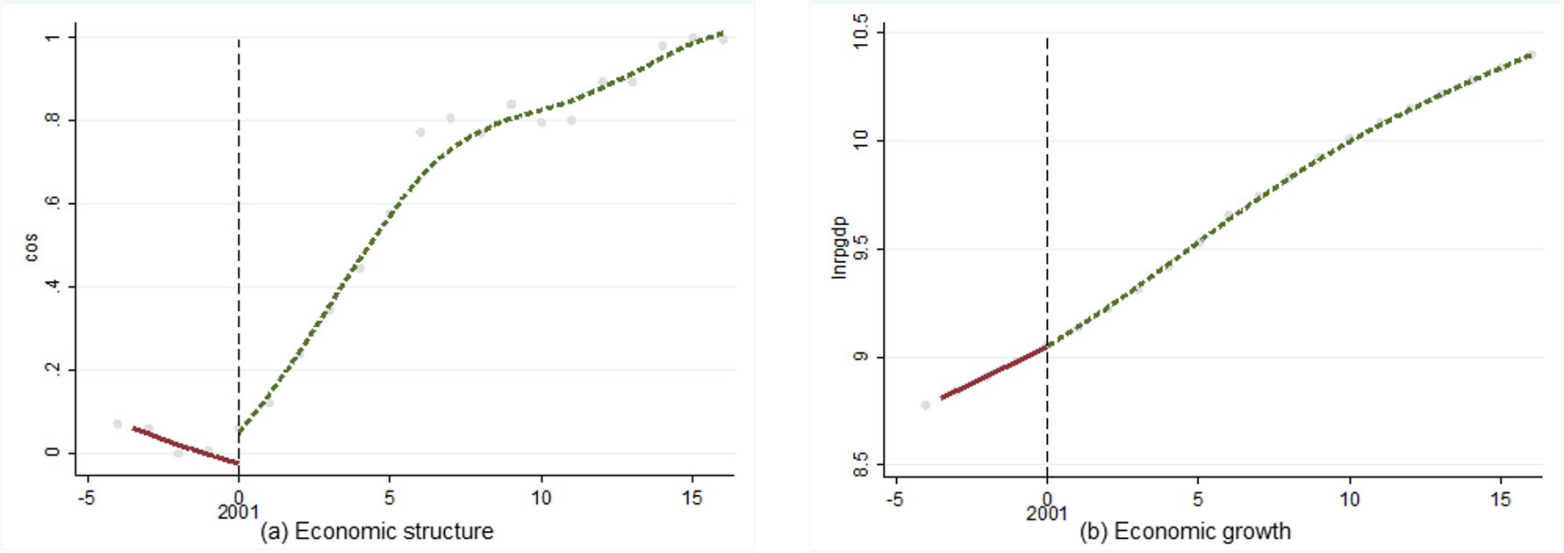
Discontinuity point in 2001 (lower-middle-income).

**Fig 5:**
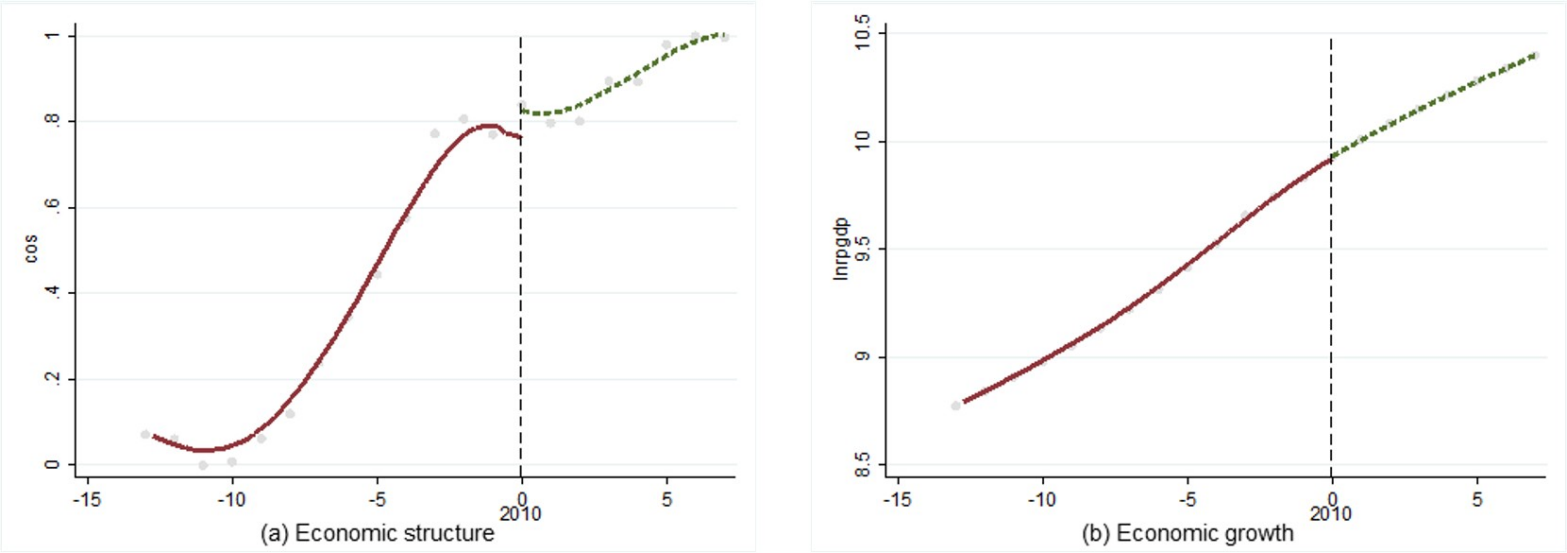
Discontinuity point in 2010 (upper-middle-income stage).

Regression Discontinuity Design was first proposed by Thistlethwaite and Campbell [41]. As a method based on natural experiments, it is mostly used to test the effect of policy implementation. “Entering the middle-income stage” is not a realistic policy, but as an objective, and it may have a real impact on economic operation. Therefore, this section drew on this idea to conduct preliminary fact-checking analysis. We took 2001 (lower-middle-income stage) and 2010 (upper-middle-income stage) as the time periods. If there is a significant jump in economic structure or economic growth, so-called discontinuity point, then the difference may be caused by the fact that China has entered the middle-income stage. A two-stage analysis of economic structure and economic growth was conducted as follows (Figs 4 and 5).

The results showed that economic structure produced an obvious discontinuity point in lower-middle-income stage, but economic growth did not; the situation was the same in the upper-middle-income stage. Thus, influenced by the fact of China entered the middle-income stage, the development of economic structure produced obvious differences, but economic growth did not been affected.

### 4.2 The measure about the imbalance of economic structure

The Principal Component Analysis was used to compute the imbalance index. We built the measurement of the structural imbalance based on the existing reference [42] to classify the absolute values of factor scores via Principal Component Analysis of six sub-structures and comprehensive structure, in order to assess the differences in structural imbalances (Table 2).

**Table 2:** The measurement of the structural imbalance.

Imbalance index	[0,0.25)	[0.25,0.5)	[0.5,0.75)	[0.75,1]
Degrees of structural imbalance	Normal	Mild	Moderate	Severe

**Fig 6:**
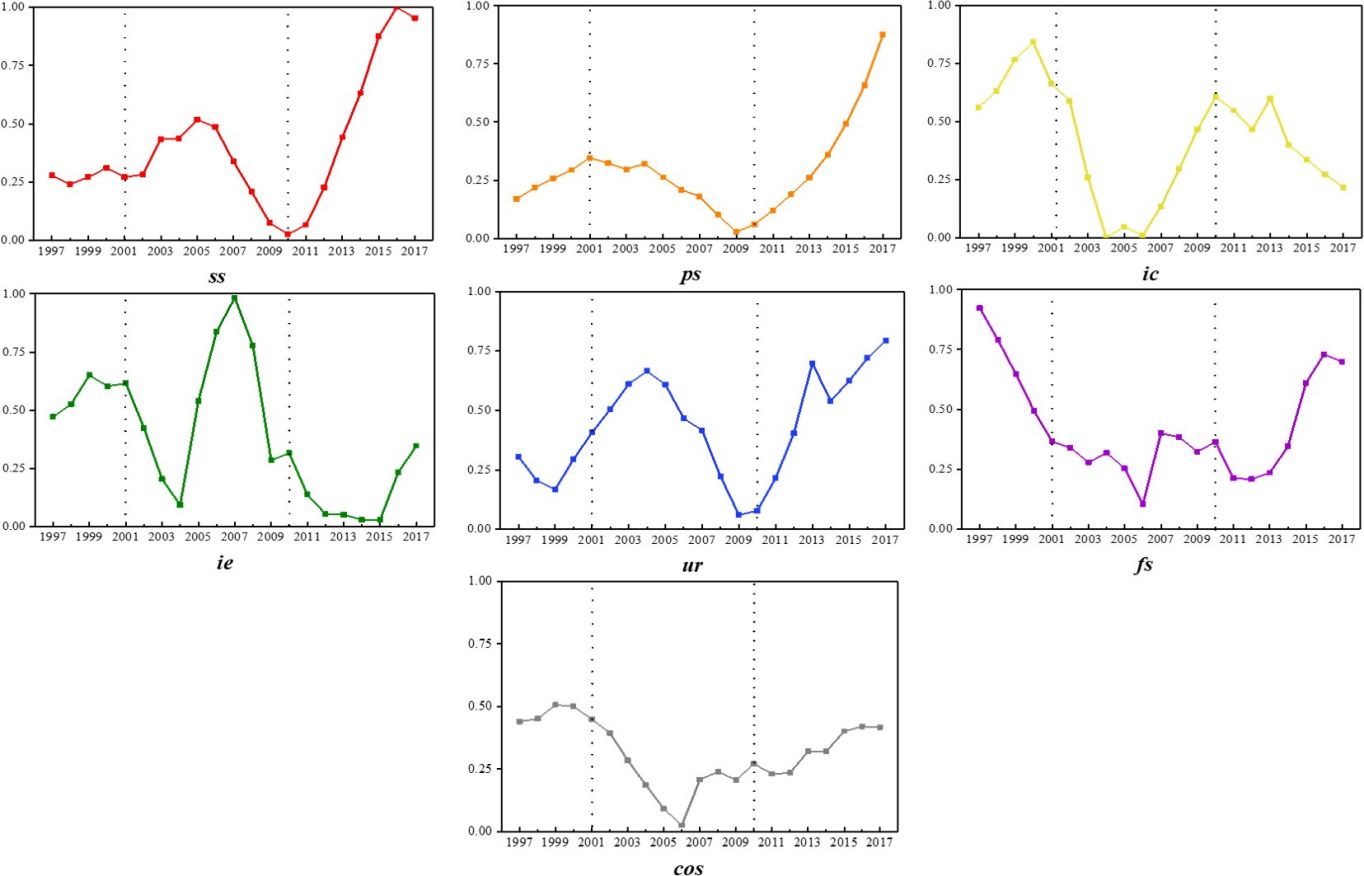
The imbalance index of economic structure.

The results (Fig 6) showed that, firstly, there were significant differences in the variation trends of the indexes. The imbalance indexes of financial structure and comprehensive structure approximately conformed to U-shape, that was, the index declined first, and then gradually increased. Sectoral structure, population structure, and urban-rural income structure approximately conformed to N-shape. Import-export structure, investment and consumption structure approximately conformed to anti-N-shape (И). Secondly, there were also significant differences in the value range of the index. Six sub-structures experienced four stages of imbalances: normal, mild, moderate, and severe. Comprehensive structure went through two stages: normal and mild. It could be seen that although the imbalances of six sub-structures were complex and evident, the imbalance of comprehensive structure was relatively mild. The six aspects interacted with each other to influence comprehensive economic structure.

### 4.3 Factual tests of the impact of different regional economic structures on economic growth

China is a country in transition with unbalanced regional development. The endowments of factors and economic structures differ across China’s various regions which are at different stages of development. The report of the 19th National Congress of China stated that “strengthening measures to promote the development of the western region to form a new pattern, deepening reforms and accelerating the revitalization of old industrial bases such as the Northeast, giving full play to promote the rise of the central region, let innovation take the lead in achieving optimized development in the eastern region, and establishing a more effective new regional coordinated development mechanism.” Therefore, this study divided 286 prefecture-levels into four regions: east, center, west, and northeast based on the listing of the four major economic regions announced by the National Bureau of Statistics of China (http://www.stats.gov.cn/ztjc/zthd/sjtjr/dejtjkfr/tjkp/201106/t20110613_71947.htm).

#### Regression model

The preliminary measures in sub-section 4.2 provided a credible basis for regression tests. We described the variation trend of the structure as U-shape, N-shape, and anti-N-shape. These trends were likely to fit the quadratic and cubic function forms. The prefecture-levels indicators were used in the test.

The econometric models were given as follows.


$$\begin{array}{l} lnrpgdp_{i,t}=\beta_{0}+\sum_{\alpha =1}^{3}\beta_{1\alpha }ss_{{}_{i,t}}^{\alpha }+\sum_{\alpha =1}^{3}\beta_{2\alpha }ps_{{}_{i,t}}^{\alpha }+\sum_{\alpha =1}^{3}\beta_{3\alpha }ic_{{}_{i,t}}^{\alpha }+\sum_{\alpha =1}^{3}\beta_{4\alpha }ie_{{}_{i,t}}^{\alpha }\\ \;+\sum_{\alpha =1}^{3}\beta_{5\alpha }ur_{{}_{i,t}}^{\alpha }+\sum_{\alpha =1}^{3}\beta_{6\alpha }fs_{{}_{i,t}}^{\alpha }+\eta X_{i,t}+u_{i}+q_{t}+\varepsilon_{i,t} \end{array}$$ (37)

$$lnrpgdp_{i,t}=\beta_{0}+\sum_{\alpha =1}^{3}\gamma_{1\alpha }cos_{{}_{i,t}}^{\alpha }+\eta X_{i,t}+u_{i}+q_{t}+\varepsilon_{i,t}$$ (38)

Our previous work tested the significance of differences in coefficients, so the fixed-effects models were used for regression testing. Control variables (*X*) included government size (*go*), capital size (*ca*), and city size (*ci*). See S1 File for more details of control variables.

#### Benchmark results

Table 3 reported the summary of the results of Eq (37). Table 4 reported the results of Eq (38). The results of all cities were that all cubic coefficients were significant. Among them, the cubic coefficient of *fs* was positive, and the others were negative. The results in other regions could also be from the tables above.

**Table 3:** Results of Eq (37) (dependent variable: *lnrpgdp*).

	All cities	Eastern cities	Central cities	Western cities	Northeastern cities
*ss* ^*3*^	-5.425***	-5.739***	-5.019**	-4.486***	-5.857***
	(0.476)	(0.782)	(1.765)	(0.914)	(1.379)
*ss* ^*2*^	7.565***	7.624***	7.709***	6.279***	7.458***
	(0.594)	(1.026)	(1.826)	(1.142)	(1.395)
*ss*	-3.002***	-2.905***	-3.104***	-2.250***	-3.107***
	(0.204)	(0.368)	(0.510)	(0.409)	(0.425)
*ps* ^*3*^	-0.553*	-3.956***		-1.841***	-3.272***
	(0.296)	(0.865)		(0.731)	(0.739)
*ps* ^*2*^	1.307***	6.746***	0.491***	4.099***	3.798***
	(0.496)	(1.353)	(0.155)	(1.173)	(0.968)
*ps*	-0.519**	-3.320***	-0.231*	-2.300***	-1.045***
	(0.226)	(0.674)	(0.137)	(0.590)	(0.400)
*ic* ^*3*^	-1.045***				
	(0.36)				
*ic* ^*2*^	0.978***	1.194***		-0.519***	-0.638***
	(0.415)	(0.336)		(0.201)	(0.221)
*ic*	0.294*	-0.221	0.188*	1.019***	1.117***
	(0.136)	(0.203)	(0.101)	(0.204)	(0.187)
*ie* ^*3*^	-1.410***	-42.37***	-2.213***		
	(0.513)	(17.22)	(0.882)		
*ie* ^*2*^	1.417***	22.83***	2.305***		
	(0.583)	(8.094)	(0.967)		
*ie*	-0.330***	-3.172***	-0.394	-0.103	0.015
	(0.154)	(0.786)	(0.244)	(0.071)	(0.095)
*ur* ^*3*^	-1.860***				
	(0.567)				
*ur* ^*2*^	3.491***			-0.875***	0.417**
	(1.071)			(0.383)	(0.173)
*ur*	-1.716***	0.201*	0.451***	1.193***	-0.451*
	(0.649)	(0.121)	(0.108)	(0.482)	(0.241)
*fs* ^*3*^	1.624***	3.578***			-12.75***
	(0.470)	(0.661)			(3.801)
*fs* ^*2*^	-1.573***	-4.791***	-1.290***	0.591***	9.216***
	(0.558)	(0.911)	(0.403)	(0.290)	(2.682)
*fs*	-0.180	1.288***	0.245	-1.073***	-2.901***
	(0.181)	(0.319)	(0.237)	(0.223)	(0.595)
Control variables	Yes	Yes	Yes	Yes	Yes
City fixed effects	Yes	Yes	Yes	Yes	Yes
Year fixed effects	Yes	Yes	Yes	Yes	Yes
Observations	6,006	1,827	1,680	1,722	777
Num. of cities	286	87	80	82	37

(i) Standard errors were in parentheses. (ii) Significance levels:

*** 1%

** 5% and

* 10%. (iii) Only coefficients significant at 10% were reported.

**Table 4:** Results of Eq (38) (dependent variable: *lnrpgdp*).

	All cities	Eastern cities	Central cities	Western cities	Northeastern cities
*cos* ^*3*^	-40.900***	-51.118***	-81.421***		-96.632***
	(8.619)	(14.830)	(29.812)		(16.860)
*cos* ^*2*^	37.584***	44.401***	83.251***		91.101***
	(8.7001)	(15.896)	(27.838)		(15.867)
*cos*	-10.719***	-11.909***	-26.119***	0.342*	-28.076***
	(2.876)	(5.576)	(8.535)	(0.204)	(4.924)
Control variables	Yes	Yes	Yes	Yes	Yes
City fixed effects	Yes	Yes	Yes	Yes	Yes
Year fixed effects	Yes	Yes	Yes	Yes	Yes
Observations	6,006	1,827	1,680	1,722	777
Num. of cities	286	87	80	82	37

(i) Standard errors were in parentheses. (ii) Significance levels:

*** 1%

** 5% and

* 10%. (iii) Only coefficients significant at 10% were reported.

#### Further discussion

Based on the properties of the power functions, especially the cubic function, we made a further investigation of the benchmark results. We calculated the “local(relative) maximum/minima points”. Note that we were concerned with the values of independent variables at the local(relative) maximum/minima, rather than local(relative) maximum/minima themselves. Those values were named “local(relative) maximum/minima points” in mathematics. So we called them “extremum points” for short, of the regression equations in the benchmark results by the constrained optimization method. In addition, we drew the increase and decrease of the function value.

**Fig 7:**
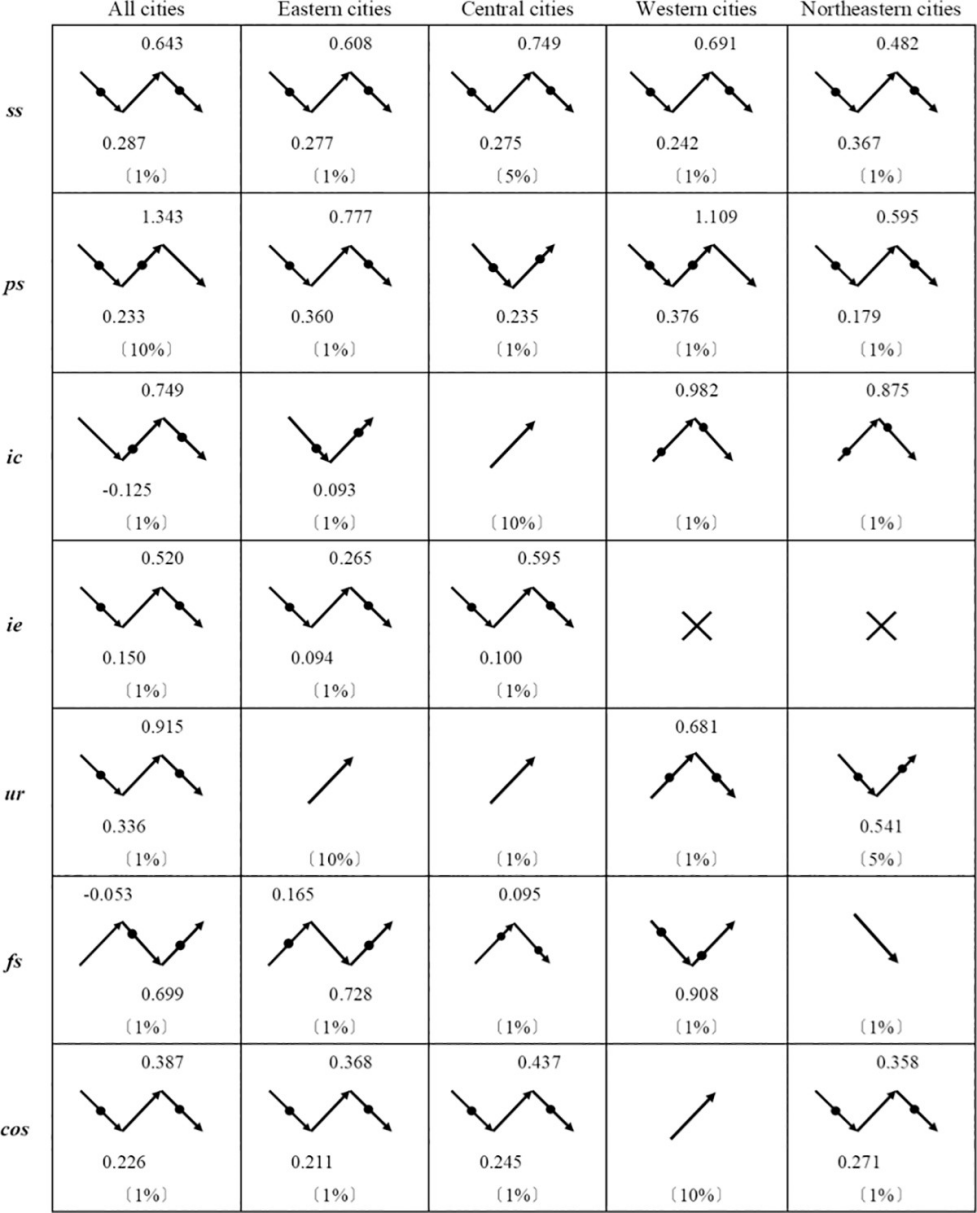
Trends of structure on economic growth according to empirical results.

Fig 7 showed the patterns of structure on economic growth nationwide and in the four regions, as well as changes in the impact of six sub-structural and comprehensive structural indexes on economic growth. Note that numbers were the values of local(relative) maximum/minima points; significance levels were in parentheses; the symbol × meant that it was not significant.

The effect of sectoral structure on the economic growth in the east, center, west, and northeast underwent a change of suppression-promotion-suppression. Judging from the values of the extreme points, after entering the middle-income stage, the inhibitory effect of the western sectoral structure on economic growth turned to the promotion effect, followed by the eastern, central, and northeast. But the restraint first appeared in northeast and then began to restrain the economic growth of the east, west, and central regions in turn.

Population structure promoted economic growth after a short-term suppression with lagged economic growth of the country and the central and western regions, indicating that the demographic dividend decline in the central and western regions is not serious; the eastern and northeastern regions experienced the suppression-promotion-inhibition changes, of which the maximum value for the eastern was close to 1, indicating that the suppression of economic growth in the east did not occur in the initial and middle stages of the demographic transition. Although the demographic dividend declined, it did not completely disappear in the east. The maximum point in the northeast was relatively small, indicating that the demographic dividend in the northeast was exhausted.

Although the regression results showed different effects of the investment and consumption structure on the economic growth of the country, west, and northeast, they had similarities. The national minimum point was less than 0, indicating that the investment and consumption structure promoted economic growth significantly during this period, but then negative effects would follow. The situation in the west and northeast were the same; the eastern minimum point was close to 0, indicating after a short period of adjustment, investment and consumption structure continued to promote economic growth in the east; the effect was always positive in the central region. Therefore, in the eastern and central regions, investment was a stronger driving force for economic growth than consumption. In the western and northeastern regions, there would be a lack of investment promotion in the later stages of the transition.

The effects of import-export structure on economic growth went from negative to positive and then negative in national, western, central, and western China. The suppression of the eastern import-export structure appeared first. Eastern region was more dependent on foreign trade, so issues such as trade disputes, currency appreciation pressure, etc., caused by the change of import-export structure, might affect the eastern region first. Next, this situation occurred in the central region. The impacts in western and northeastern regions were not statistically significant.

Nationally, the impact of urban-rural income structure on economic growth was through the stages of suppressed-promoted-suppressed, in line with the general law of economic growth driven by factors of production affected by income gaps. Urban-rural income structure continued to promote economic growth in the east and central regions, from promotion to suppression in the west and suppression to promotion in the northeast. This result showed that the factors of production affected by the income gap in the east, central, and northeast could still drove economic growth. The situation of the urban-rural income gap was not serious, while the expansion of the urban-rural income gap in the west had a negative effect on economic growth.

The effect of financial structure on economic growth went from positive to negative and then positive in eastern region, but opposite in central, western, and northeastern regions. Problems such as the global economic crisis, large-scale foreign exchange reserves, etc., put financial structure at risk of imbalance. The eastern region had a high degree of financial development and a relatively reasonable financial structure, so it could still promote economic growth after a short period of adjustment. However, the financial developments of central, western, and northeastern regions were lagging behind, and their financial structures were fragile. It was difficult to get rid of the adverse effect of the financial structural imbalance on economic growth.

In general, the effects of comprehensive structure on economic growth went from negative to positive and then negative in national, eastern, central, and northeastern China. Comprehensive structure of western region could still promote economic growth but the impact was small.

These results might indicate a signal of China’s industry, investment and consumption drivers weakening; a signal of the demographic dividend diminishing; a signal of the international trade challenges; a signal of inadequate financial development. The pattern of income disparity driving economic growth should be changed. Comparing and summarizing the above results, the structural drivers of regional economic growth were different since entering the middle-income stage. There were their own strong drives and shortcomings. All kinds of “contradictions” caused “throes”. By taking the initiative, China did not face the risk of economic stagnation, but it would be urgent to accelerate the optimization and upgrading of the economic structure.

## 5. Robustness tests

Comprehensive economic structure constructed by six sub-structures might be interrelated through the various structures and the subjectivity of the weight of the index. To avoid the accidental conclusion of the research, we further tested the following three aspects to verify the robustness of previous regression results.

### 5.1 GMM: Eliminating continual effects and possible endogeneities

Economic phenomena are continual, and past economic growth may have an impact on the current period. So the lagging one period and lagging two periods of economic growth (*L1*.*lnpegdp*, *L2*.*lnpegdp*) were included in the explanatory variables. However, random process, heteroskedasticity, and serial correlation problems arose in the new regression model. Therefore, the two-stage generalized moment method (GMM) was used as an alternative model to test the robustness of the model by setting the maximum lags to 2. We tested the Eq (38) only. The reason was that comprehensive structure contained all the characteristics of six-aspect structures and the variables were simplified greatly, which ensured the validity of the instrumental variables (IVs were *L1*.*go*, *L1*.*ca*, and *L1*.*ci*).

It could be seen from Table 5 that *L1*.*lnpegdp* nationwide passed the test, and the *L2*.*lnpegdp* did not pass the test, indicating that the sustained impact of economic growth was universal and had a short duration. Comparing the regression results in Table 4, the sign and significance of the regression coefficients were exactly the same as those of the benchmark regression, but there were differences in the significance levels: the significance levels in the central and northeast regions decreased to 5%, and the significance levels in the western region increased to 5%. Therefore, adding the lags and using different methods to estimate did not change the original conclusion.

**Table 5:** GMM regression of Eq (38) (dependent variable: *lnrpgdp*).

	All cities	Eastern cities	Central cities	Western cities	Northeastern cities
*L1*.*lnpegdp*	0.407***	0.384***	0.720***	1.094***	1.177***
	(0.163)	(0.098)	(0.149)	(0.087)	(0.171)
*L2*.*lnpegdp*	0.053	-0.082	0.239*	0.005	-0.110
	(0.058)	(0.059)	(0.067)	(0.640)	(0.135)
*cos* ^*3*^	-155.25***	-349.257***	-593.06**		-51.26*
	(55.427)	(130.221)	(242.80)		(27.89)
*cos* ^*2*^	160.842***	380.692***	561.35**		43.05*
	(55.560)	(143.418)	(220.080)		(24.16)
*cos*	-49.519***	-130.394***	-166.363**	1.425**	-13.24**
	(17.527)	(49.586)	(64.322)	(0.678)	(6.579)
Control variables	YES	YES	YES	YES	YES
AR(1) test	-3.14	-2.26	-2.31	-4.87	-1.87
	[0.002]	[0.024]	[0.021]	[0.000]	[0.061]
AR(2) test	-1.15	-0.27	-0.12	-1.04	-1.38
	[0.250]	[0.789]	[0.905]	[0.298]	[0.167]
Hansen test	7.11	3.59	10.33	3.65	8.42
	[0.418]	[0.309]	[0.171]	[0.302]	[0.297]
Num. of IVs	15	15	15	9	15
Observations	6,006	1,827	1,680	1,722	777
Num. of cities	286	87	80	82	37

(i) For variables, standard errors were in parentheses; and for statistics, *p* values were in parentheses. (ii) Significance levels:

*** 1%

** 5% and

* 10%. (iii) Some studies usually report Sargan test, while Hansen test is more robust. However, too many IVs will make the *p* value of Hansen test equal to 1 significantly and lose the explanatory effect, so the valid condition of IVs is that the *p* value is greater than 0.1 but cannot be equal to 1 significantly. (iv) Only coefficients significant at 10% were reported.

### 5.2 Reset synthesized weight: Considering logical endogenous relation

The 19th National Congress of China put forward the goal of “focusing on accelerating the construction of an industrial system for the coordinated development of the real economy, technological innovation, modern finance, and human resources”, and for the first time, modern finance was included in the industrial system. New Structural Economics believes that the factor endowment of one economy and its structure is given at a specific stage of development [43]. Factor endowment determines the optimal sectoral structure and it is inseparable from the support of financial structure, which adapted to the specific stage. Therefore, financial structure is produced in sectoral structure and their interlocking is based on the theoretical framework. It may affect the synthesis of comprehensive structure, thus affecting the empirical results. We built the new comprehensive structure (*NEWcos*) by resetting synthesized weight: sectoral structure turned to 1/4, financial structure turned to 1/12, and other four remained 1/6. We tested the Eq (38).

The results (Table 6) of the significance levels and signs of coefficients almost agreed to the empirical results above. Therefore, the empirical results were acceptable.

**Table 6:** Significant results summary of Eq (38) by the new weight (dependent variable: *lnrpgdp*).

	All cities	Eastern cities	Central cities	Western cities	Northeastern cities
*NEWcos* ^*3*^	-45.361***	-47.890***	-47.485**		-95.758***
	(8.628)	(14.759)	(24.712)		(16.263)
*NEWcos* ^*2*^	44.087***	44.062***	53.766**		91.314***
	(8.810)	(16.172)	(23.77)		(15.323)
*NEWcos*	-13.447***	-12.697**	-18.538**	0.478**	-28.334***
	(2.942)	(5.755)	(7.499)	(0.200)	(4.754)
Control variables	Yes	Yes	Yes	Yes	Yes
City fixed effects	Yes	Yes	Yes	Yes	Yes
Year fixed effects	Yes	Yes	Yes	Yes	Yes
Observations	6,006	1,827	1,680	1,722	777
Num. of cities	286	87	80	82	37

(i) Standard errors were in parentheses. (ii) Significance levels:

*** 1%

** 5% and

* 10%. (iii) Only coefficients significant at 10% were reported.

### 5.3 SDM: Verifying spatial autocorrelation effect

In the study of regional economic development, due to the existence of the “First Law of Geography” [44], the issue of spatial autocorrelation between different regions has received increasing attention. In order to verify whether the spatial autocorrelation has an impact on the regression results, we collected 286 prefecture-levels data for latitude and longitude, calculated Moran’s *I* from 1997 to 2017 (see S1 File), and examined the spatial dependence of economic growth nationwide by a Spatial Dubin Model (SDM) considering the hysteresis of independent variables.

Table 7 summarized the results of SDM. For the average effect, the results of SDM were completely consistent with empirical results above for the signs, the trends of change, and the levels of significance. Further comparison with Table 3 showed that the investment and consumption structure actually promoted then suppressed, which also agreed to the results in Table 7. For the spatial hysteresis effect, only sectoral structure’s impact was positive and significant at 1% level, indicating that sectoral structure of a city would play a certain role in promoting the economic growth of surrounding cities. It might be due to the most obvious stickiness and radiation effects of regional sectoral structures. In addition, the Moran’s *I* did not exceed 0.45 and the fluctuation was relatively low, indicating that the spatial correlation difference did not change significantly in 21 years. Therefore, the regression results were robust.

**Table 7:** SDM regression of Eqs (37) and (38) (dependent variable: *lnrpgdp*).

	(37)		(38)	
	Average effect	Hysteresis effect	Average effect	Hysteresis effect
*ss* ^*3*^	-4.194***	5.198***		
	(0.944)	(1.723)		
*ss* ^*2*^	5.871***	-6.877***		
	(1.187)	(2.244)		
*ss*	-2.364***	3.032***		
	(0.416)	(0.740)		
*ps* ^*3*^	-1.185*	0.101		
	(0.633)	(1.007)		
*ps* ^*2*^	2.308**	-0.902		
	(0.916)	(1.433)		
*ps*	-1.01**	0.859		
	(0.417)	(0.626)		
*ic* ^*3*^				
*ic* ^*2*^	-0.643***	-0.113		
	(0.223)	(0.348)		
*ic*	1.074***	-0.147		
	(0.201)	(0.294)		
*ie* ^*3*^	-3.052***	7.858		
	(1.185)	(7.676)		
*ie* ^*2*^	3.090**	-6.984		
	(1.422)	(6.409)		
*ie*	-0.676**	1.366		
	(0.342)	(1.088)		
*ur* ^*3*^	-1.914**	1.035		
	(0.771)	(1.513)		
*ur* ^*2*^	3.411**	-1.916		
	(1.335)	(2.832)		
*ur*	-1.598**	0.979		
	(0.709)	(1.758)		
*fs* ^*3*^	1.176***	-0.458		
	(0.424)	(0.923)		
*fs* ^*2*^	-1.116**	-0.345		
	(0.496)	(1.040)		
*fs*	-0.212	0.547*		
	(0.167)	(0.312)		
*cos* ^*3*^			-59.925***	36.630
			(19.661)	(38.525)
*cos* ^*2*^			57.710***	-42.598
			(19.137)	(36.323)
*cos*			-17.574***	16.265
			(6.101)	(11.303)
Control variables	YES		YES	
R-square	0.4674		0.4597	
Observations	6,006		6,006	
Num. of cities	286		286	

(i) Standard errors were in parentheses. (ii) Significance levels:

*** 1%

** 5% and

* 10%.

## 6. Conclusion

Focusing on the background of China’s economic growth slowdown and economic transformation in the middle-income stage, this study took the perspective of economic structures. The paper found that after entering the middle-income stage, the structural dividend tended to be exhausted, and structural imbalances restricted economic growth. There were significant differences between the structural imbalances of various sub-items and economic growth at different stages and in different regions. However, overall economic structural imbalances and economic transformation coexisted. The economy was growing in a slowdown.

Specifically, the effects of comprehensive structure on economic growth went from negative to positive and then negative in national, eastern, central, and northeastern China; comprehensive structure of western region could still promote economic growth but the effect was small. For sectoral structure, with the economy going servicizing, the suppressive effects emerged. The demographic dividend was still good in central and western regions; it was not depleted completely in eastern region; but it was severe in northeastern region. Investment and consumption structure had a stronger driving force on economic growth in eastern and central regions but the impact weakened in western and northeastern regions. For import-export structure, there were similar effects in national, eastern, central regions, but the suppressive effect appeared first in eastern region with higher dependency on foreign trade. For urban-rural income structure, eastern, central, and northeastern regions were still in the growth model driven by urban-rural income gap; however, the widening urban-rural income gap stifled western growth. For financial structure, it could still promote economic growth after a short period of adjustment in the eastern region given the more developed financial system; but it had a negative effect in other regions.

After entering the middle-income stage, accumulating China’s structural bonus over a long period was unable to adapt to the transformation from quantity growth to quality growth of its economy. Predictably, the development of China’s economic structure was not optimistic. There would be crises that imbalance grows, and deviates from economic growth. Therefore, China should adhere to improve and upgrade economic structure in the long term, while the policies should be tailored to local conditions. In particular, China should persist in expanding domestic demand; establish the leading position of sectoral structure optimization in the process of structural adjustment; deepen the reform of the investment and financing system; and actively respond to the challenge of population fertility.

## Supporting information

S1 File (DOCX)
