# Supplementary material for: The new evidence of China’s economic downturn: From structural bonus to structural imbalance
Source: PLoS One. 2021 Sep 23;16(9):e0257456. doi: 10.1371/journal.pone.0257456 (PMC8459991; doi:10.1371/journal.pone.0257456)
Supplement: S1 File — (DOCX) [file pone.0257456.s001.docx]

**Data processing**

**Industry comparison index** is defined as follow.

$$II=\frac{(\frac{agP}{P})/(\frac{agZ}{Z})}{(\frac{nagP}{P})/(\frac{nagZ}{Z})}$$

Where variables *agP*, *nagP*, *P*, *agZ*, *nagZ*, and *Z* denote, respectively, agriculture output, non-agriculture output, total output, agriculture employment, non-agriculture employment, and total employment.

**Economy servicizing index** is defined as the ratio of services output to manufacturing.

**Unit GDP energy consumption** is defined as the ratio of total energy consumption (tons of standard coal) to GDP (ten thousand CNY).

**Children’s dependency ratio** is defined as the ratio of the population under age 15 to population aged 15-64.

**Elderly dependency ratio** is defined as the ratio of the population over age 64 to population aged 15-64.

**Gini coefficient of education** is defined as follows.

$$EDU_{i}=\frac{EduY_{i}\cdot PN_{i}}{\sum_{i=1}^{5} EduY_{i}\cdot PN_{i}}$$

Where *i=*1,2,3,4,5 denote, respectively, the education level is illiterate & semi-literate, primary school, junior high school, high school, junior college & above. The respective years of education, denoted by the variable *EduY_i_* are 0, 6, 9, 12, 16 years. Variable *PN_i_* denotes educated population. So *EduY_i_* ∙ *PN_i_* denotes the cumulative achievement of each level. Variable *EDU_i_* denotes the cumulative achievement ratio of each level.

$$POE_{i}=\sum_{i=1}^{5} EDU_{i}$$

Where variable *POE_i_* denotes the total cumulative achievement ratio of education.

$$EG=1-\frac{1}{5}(2\sum_{i=1}^{5-1} POE_{i}+1)$$

Where variable *EG* denotes the Gini coefficient of education.

**Total population participation rate** is defined as the ratio of employment to total population.

**Theil index of urban-rural income gap** is defined as follow.

$$T=\sum_{i=1}^{2} [(\frac{INC_{i}}{INC})\ln(\frac{INC_{i}/INC}{Z_{i}/Z})]$$

Where variables *INC_i_*, *INC*, *Z_i_*, and *Z* denote, respectively, regional income, total income, regional income, and total income.

**Ratio of direct financing to indirect** is defined as follow.

$$ROF=\frac{SMV+OCB}{BCB}$$

Where variables *SMV*, *OCB*, and *BCB* denote, respectively, stock market value, outstanding balance of corporate bonds, and bank credit balance.

**Napierian logarithm of real per capita GDP.** 1997 is as the base period. We calculate the GDP deflator by the GDP index (in the provincial and municipal level) at first. Then we use the GDP deflator to calculate the per capita GDP of all cities (matching the provinces or municipalities that they belong to) and take the Napierian logarithm.

**Government size** is defined as the ratio of financial expenditure to GDP.

**Capital size** is defined as the ratio of investment in the fixed assets to GDP.

**City size** is defined as population density of city.

**Standardization of variables**

The units of variables are different, so they cannot be compared with each other directly. Here is a standardized way for original data.

$$X(s)_{i}=\frac{X_{i}-minX_{i}}{maxX_{i}-minX_{i}}$$

Where *X*(*s*)*i*, *X_i_*, *minX_i_*, and *maxX_i_* denote, respectively, standardized value, original value, the minimum value of all values in the same variable, and the maximum value of all values in the same variable.

**Moran’s *I* of real per capita GDP in 286 cities in China (1997-2017)**

Moran’s *I* is defined as follow.

Where *X_i_* denotes the observed value of region *i* (that is real per capita GDP). , . Where *W* and *W_ij_* denote the spatial weight matrix and its element. The longitude and latitude data of 286 cities are collected in Google Earth and then we calculate the *W* by xy2cont function in MATLAB.

The value range of Moran’s *I* is [-1,1]. A value greater than 0 indicates a positive spatial autocorrelation. The Moran’s *I* of real per capita GDP in 286 cities of China (1997-2017) are shown as follows.

**Table S1. Moran’s *I* of real per capita GDP in 286 cities of China (1997-2017).**

| Year | Moran’s *I* | Year | Moran’s *I* | Year | Moran’s *I* |
| --- | --- | --- | --- | --- | --- |
| 1997 | 0.332 | 2004 | 0.390 | 2011 | 0.37 |
| 1998 | 0.345 | 2005 | 0.354 | 2012 | 0.363 |
| 1999 | 0.324 | 2006 | 0.368 | 2013 | 0.359 |
| 2000 | 0.279 | 2007 | 0.383 | 2014 | 0.377 |
| 2001 | 0.285 | 2008 | 0.383 | 2015 | 0.384 |
| 2002 | 0.334 | 2009 | 0.384 | 2016 | 0.375 |
| 2003 | 0.346 | 2010 | 0.394 | 2017 | 0.357 |
